# Characterization and phylogenetic analysis of the complete mitochondrial genome from Rock Scallop (*Crassadoma gigantea*) using next-generation sequencing

**DOI:** 10.1080/23802359.2018.1483752

**Published:** 2018-07-27

**Authors:** Dejie Liao, Ying Zhou, Jingou Tong, Shanmao Cao, Xiaomu Yu, Beide Fu, Dazuo Yang

**Affiliations:** a Liaoning Shellfish Fine Breed Breeding Engineering Technology Research Center, Dalian Ocean University, DaLian, China;; b University of Chinese Academy of Sciences, Beijing, China;; c State Key Laboratory of Freshwater Ecology and Biotechnology, Institute of Hydrobiology, The Chinese Academy of Sciences, Wuhan, China;; d Key Laboratory of Marine Bio-resources Restoration and Habitat Reparation in Liaoning Province, Dalian Ocean University, Dalian, China

**Keywords:** Rock scallop, mitochondrial genome, phylogenetic relationship

## Abstract

In this study, the complete 18,495 bp mitochondrial genome was determined from Rock scallop (*Crassadoma gigantea*) using next-generation sequencing technology. The complete mitochondrial genome contained 12 protein-coding genes (PCGs), 2 ribosomal RNA genes, 23 transfer RNA genes, without *ATP8* and *D-loop*, which was similar with most mitochondrial genomes of marine bivalve molluscs. Gene annotations, including gene order, genetic code, start and stop codons and codons bias, were identified. Phylogenetic tree was constructed using Neighbor-Joining (NJ) method based on the PCGs showed the present species clustered within the Pteriomorphia clade. This work should be of importance not only for the better understanding of the relationships within Pectinidae, but also for the development of useful genetic markers in Rock scallop aquaculture and restoration efforts.

Rock scallop (*C. gigantea*), which belongs to Mollusca, Lamellibranchia, Pterioida, was native to North America Pacific coast (Beitler et al. 1991). There are several valuable characteristics making rock scallop favored in the international market, including fast growth, strong resistance and delicious meat. The rock scallop was introduced to China to improve the stability and solve the problem of lacking varieties in scallop aquaculture. To date, the studies on rock scallops are mainly focused on physiology, ecology, production and aquaculture (MacDonald and Bourne 1989; Culver et al. 2000, 2006; Cao et al. 2017), while the genetics background was rarely reported. Here, the complete mitochondrial genome (mitogenome) of rock scallop was obtained by high-throughput sequencing using an Illumina HiSeq 2500 with paired-end 150 PE rapid run chemistry.

The specimen with 2 years old was collected from the Longwangtan, Bohai sea area (Dalian, Liaoning, China), which was derived from Vancouver, Canada. Similar to other bivalves, the mitochondrial genome of *C. gigantea* was a circular molecule with 18,495 bp in length and had been deposited in GenBank with accession number (MH016739). It contained 12 protein-coding genes (PCGs), 2 rRNAs, 23 tRNAs and non-coding regions (NCRs). Like most mitochondrial genomes of marine bivalve molluscs, the protein-coding gene *ATP8* had not been identified in *C. Gigantea*. Within the whole mitochondrial genomes of *C. gigantea*, three largest non-coding regions were located between *cox1* and *nad4L* (1173bp), *cox2* and *nad2* (637bp), and *trnN-aac* and *trnV-gta* (487bp), respectively. Furthermore, different start codons of protein-coding genes were identified by careful manual inspection, though they were highly biased towards ATG. For most genes, the stop codons (TAG) were identified relative completely.

The base compositions of the *C. gigantea* mitochondrial genome were A = 21.19%, C = 15.24%, G = 28.75%, and T = 34.82%, with an overall A + T content of 56.02%. To investigate the nucleotide bias, the AT and GC skews were calculated as (A-T)/(A + T) and (G-C)/(G + C), respectively (Puslednik et al. 2008). The AT and GC skews for the mitochondrial genome of *C. gigantea* were –0.243 and 0.309, respectively, indicating the occurrence of more G and T than C and A.

To future study the genetic relationship of *C. gigantea* with other scallops and the phylogenetic position in Pteriomorphia, additional 19 complete mitochondrial genomes were selected for phygenetic tree reconstruction, which were downloaded from GenBank database (Figure 1). Within Pteriomorphia, *C*. *gigantea* and *Mizuhopecten yessoensis* were in a clade sister to *Chamys farreri*, indicating a closer relationship between *C*. *gigantea* and *M*. *yessoensis*, which was similar with previous results revealed by short mitochondrial and nuclear gene fragments (Ballard et al. 2004).

**Figure 1.**
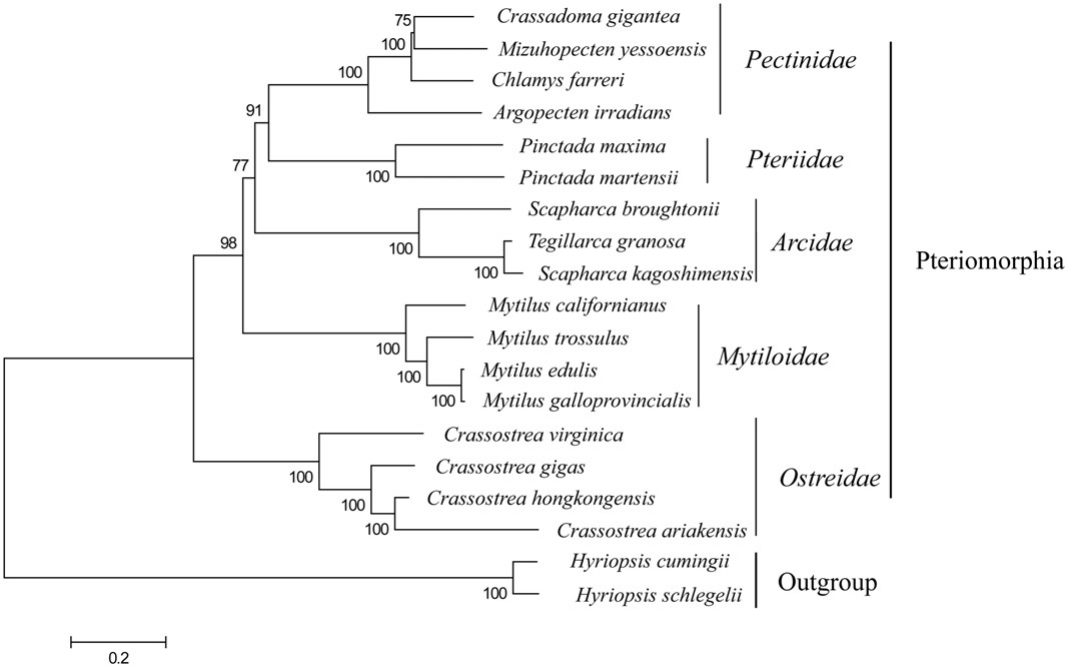
Neighbor Joining (NJ) trees based on the concatenated amino acid sequences of the PCGs.
